# Supplementary material for: Are carbon emissions trading and green financial instruments synergistic? -Comprehensive quantitative research based on content analysis
Source: PLoS One. 2024 Mar 7;19(3):e0298601. doi: 10.1371/journal.pone.0298601 (PMC10919601; doi:10.1371/journal.pone.0298601)
Supplement: S1 Data — (ZIP) [file pone.0298601.s001.zip › Data/地级市Z_F工作报告.zip!/╕╩╦α/╬Σ═■╩╨/└┤╫╘╣1⁄2╓┌║┼í╛┬φ┐╦╩2╛▌═°í┐_2014.docx]

2013年工作回顾

今年以来，面对复杂多变的经济形势和艰巨繁重的改革发展稳定任务，我们在省委、省政府和市委的坚强领导下，深入学习贯彻党的十八大、十八届三中全会精神，按照省第十二次党代会和市委三届五次全委（扩大）会的决策部署，围绕实现“五年翻番”和与全省全国同步进入全面小康社会目标，坚持以发展凝聚人心、用项目衡量政绩，不拘一格用人才、无中生有抓项目，竭力实施城乡融合、工业强市和生态立市战略，解放思想、改革创新、自加压力、真抓实干，全力推动科学发展、转型跨越、赶超进位、富民强市、构建和谐，全市呈现出经济较快增长、结构不断优化、效益显著提升、生态持续好转、民生有效改善、社会大局和谐稳定的良好态势。市三届二次人代会确定的各项目标任务可全面完成。预计全市实现生产总值401亿元，同比增长14.5%；全社会固定资产投资689.3亿元，增长35%，其中规模以上562亿元，增长35%；全部工业增加值131亿元，增长20%，其中规模以上110亿元，增长20%；社会消费品零售总额123.2亿元，增长18%；公共财政预算收入18.2亿元，增长26%；城镇居民人均可支配收入17860元，增长16%；农民人均纯收入7178元，增长17%；居民消费价格指数涨幅控制在3.5%以内。

　　一年来，主要做了九个方面的工作：

　　突出项目建设和招商引资，转型跨越发展基础不断夯实。强力推进“3341”项目工程，强化项目落地实施和调度管理，全年实施500万元以上项目1020项，其中亿元以上项目275项。签约招商引资项目315项，项目总投资1055亿元。实施招商引资项目600项，落实到位资金570亿元，增长45.85%。永古、金武、双营高速公路和古浪新井至民勤红崖山水库防沙治沙生态公路建成通车，新增高速公路里程170公里。古浪县生态移民暨扶贫开发黄花滩项目水利骨干工程建成运营。民勤县城至红沙岗一级公路、石门河调蓄引水、雷台330千伏变电站等项目加快推进，天祝县南阳山片供水工程、毛藏寺水库、天祝至青海互助二级公路、天颐大道、民勤红沙岗百万千瓦级风电基地和330千伏变电站等重大项目开工建设。

　　加快构建新型工业体系，工业经济平稳较快发展。大力实施工业强市战略，实施千万元以上工业项目352项，其中亿元以上142项，预计工业固定资产投资达到263.4亿元，增长33%。发挥省级工业发展财政引导基金作用，支持重点项目建设，带动社会资本36.17亿元，新增规模以上企业45户。青啤20万千升啤酒、荣华150万吨捣固焦、鑫淼电石深加工产业链、祁连山200万吨新型干法水泥等156项重点项目建成投产；承威3000辆专用车、凯帝斯5000台电梯等重点项目加快推进；中国航天武威新能源装备制造产业园、京奥港高档饮品、莫高国际葡萄酒城等170项重点项目开工建设。新增风光发电装机容量102.5万千瓦，累计达到121.5万千瓦。找矿行动取得重大突破，稀土、铜多金属矿、石油、煤炭等有望成为新的支柱产业。工业园区完成基础设施投资17.8亿元。武威新能源装备制造产业园区认定为省级新型工业化产业示范基地，古浪工业集中区审批为省级循环经济示范工业园区。

　　深入推进城乡融合，城镇化进程不断加快。坚持工业新型化、城镇带状化、农业现代化、信息社会化协同发展，竭力实施城乡融合战略。金色大道主线路基工程全面完工，铺油99公里，新开工建设干支联接公路498.7公里。修订完善了全域武威新农村建设总体规划，批准实施武威市新城区控制性详规、荣华工业园区控制性详规和天马湖两岸控制性详规及城市设计等规划。凉州新城区建设初具规模，中心区道路框架基本形成，金熊猫公寓、卫生大厦建成使用，体育馆、博物馆、影剧院及公安、交通、水务、农林牧、能源、人社等公共建筑进展顺利。天马湖景观治理一期工程竣工，二期工程和保障性住房、城区道路、供排水、供热等项目有序推进。优化布局中心镇和重点小城镇，新建续建300户以上新型农村社区50个、1.17万户，开工建设村（社区）综合服务中心20个，产业集聚、工农互惠和城乡融合发展步伐加快。预计年内完成城乡建设投资200亿元，全市城镇化率达到33.38%，比上年提高2.5个百分点。

　　着力加强生态文明建设，生态环境持续好转。大力实施生态立市战略，推进生态屏障行动。加快水权水价改革，优化水资源配置，全市水权配置总量控制为16.5亿立方米，生活、生态、工业和农业用水配置比例为5.1∶11.5∶15.4∶68。节水型社会建设试点通过水利部正式验收。通过调整结构节水、协调景电加大调水和人工影响天气等措施，扎实推进石羊河流域后期治理，蔡旗断面过水总量达到2.26亿立方米，民勤盆地地下水开采量控制在8557万立方米。实施甘肃民勤石羊河国家湿地公园、民勤梭梭井沙化土地封禁保护区试点、石羊河流域防沙治沙及生态恢复、三北五期、退耕还林等重点项目，全面推进红水河百里生态经济长廊建设。严格落实“五禁”规定，积极推行治沙生态林承包经营，探索工业治沙和养殖治沙新模式，大规模开展义务压沙造林活动。新增治沙造林面积15.8万亩，完成人工造林51.8万亩，封育37.4万亩，义务植树1137万株，通道绿化1275公里，治沙生态林承包面积150.9万亩。

　　强力推进主体生产模式，“三农”工作取得新成效。坚持“规模化、区域性、多品种、高效益”的原则，新建设施农牧业18.8万亩、特色林果业30.1万亩。落实各项惠农资金15.1亿元。深入推进“双联富民”和“1236”扶贫攻坚行动，超额完成21个整村推进项目年度建设任务。截至12月20日，发放“双业”贷款3.64万户、49.44亿元，“双联”贷款1.78万户、12.38亿元，两项贷款总量均居全省前列。清理收回农林场226个、12.9万亩，完成“下山入川”移民2.1万人。

　　加强市场体系建设，现代服务业发展水平不断提高。甘肃黄羊河集团物流公司被认定为“全国AA级综合服务型物流企业”，万嘉国际广场、黄羊公铁联运物流中心等项目加快推进，甘肃同威（国际）能源公司武威煤炭集疏运中心、武威昊天农产品交易市场暨仓储物流中心开工建设，武威保税物流中心即将获批。新建续建商品配送中心、专业批发市场、农村集贸市场27个。新增市、区（县）级粮食储备1.3万吨，建立“放心粮油”网点39家。成功举办“中国·河西走廊第三届有机葡萄美酒节暨中国葡萄酒城首届葡萄酒节”。华夏文明传承创新区武威项目区加快建设，天马文化产业园区暨摩天轮主题公园项目加快推进，天梯山历史文化旅游景区等项目开工建设。预计全年接待游客510万人次，实现旅游业综合收入24.9亿元，增长37.1%。新增银行授信额度1000亿元，累计达到2240亿元。甘肃银行凉州区支行挂牌成立，新增保险公司市级分支机构3家、小额贷款公司8家，准金融机构发展到22家。武威农商银行、武威银行正在审批。成功发行武威债券13亿元，首批5亿元已经到位。

　　协调发展社会各项事业，民生保障水平明显提高。九年义务教育巩固率达到99%，高中阶段教育毛入学率达到90.4%，高考综合录取率达到81.3%。兰州交通大学新能源与动力工程学院专业设置达到6个，甘肃威龙葡萄酒专修学院专业设置达到5个。新改建校舍21.6万平方米、幼儿园36所。武威职业学院一期扩建工程进展顺利。引进急需紧缺人才219名，其中博士5名、硕士84名。推进省级创新型试点城市建设，区县全部通过全国县市科技进步考核。重离子治疗肿瘤中心治疗楼和配套医院主体完工，中科院近物所武威核技术应用综合产业园开工建设，甘肃武威国家农业科技园区获批，甘肃省辐照诱变育种工程实验室、武威市畜牧兽医科学研究院挂牌成立。基本公共卫生服务均等化全面推进，基本药物制度覆盖基层，县级公立医院改革试点稳步实施。加大公共财政投入力度，认真办理民生实事，省列26件实事和市列10件实事全部完成。全市城镇新增就业3.64万人、失业人员实现再就业9681人，城镇登记失业率控制在3.5%以内；安置高校毕业生1579名，应届普通高校毕业生就业率达到90.98%。城乡居民社会养老保险基础养老金补助标准由每人每月55元提高到60元。城乡低保对象人均补助标准分别提高15%和28%。农村五保对象供养省级补助标准由人均1800元提高到2000元。基本公共卫生服务补助标准由人均25元提高到30元。新型农村合作医疗和城镇居民基本医疗保险补助标准由240元提高到280元。城乡居民社会养老、城镇居民医疗、失业保险参保率分别达到98%、101.4%、98%，新农合参合率达到99%。为5100名一级重度残疾人发放护理补贴322.8万元。发放救灾及社会救助资金4.8亿元，群众突发性、临时性困难得到有效解决。改扩建市儿童福利院，床位达到350张。改扩建乡镇卫生院11个，建成村卫生室15个、乡镇及社区体育健身中心9个。农村义务教育营养改善计划实现全覆盖，惠及农村学生13.8万名。完成7000名“两后生”职业技能学历教育培训和2000名农民劳务品牌项目培训。新改建农村公路1266公里。建成凉州城区地表水供水工程，实现向城区居民和园区供水。解决了18万农村人口饮水安全问题。新增城区天然气入户8007户、农村户用沼气1000户。新建保障性住房3.45万套，建成新农宅2.82万户，改造农村危房3.05万户，实施棚户区改造1500户。

　　加强和创新社会治理，社会大局保持和谐稳定。严格落实安全生产责任制，深入开展重点行业和重点领域安全隐患排查治理专项行动，有效预防了重特大事故发生。强化产品质量、食品药品安全和物价监管，确保了市场正常供应。民兵预备役建设和双拥共建工作不断加强。“六五”普法扎实推进。

　　加快转变政府职能，发展环境进一步优化。认真落实中央“八项规定”和省委、市委“双十条”规定，深入实施“效能风暴”行动，着力推进依法行政进程，深化行政审批制度改革，市政府行政审批项目由69项减为9项，市级部门行政审批事项由453项减为230项，证照办理事项由129项减为101项。清理并登记核准执法单位449个、执法人员5883名。各类会议、文件比上年明显减少。市、区县行政权力网上公开透明运行信息系统建成投运，市公共资源交易中心累计交易各类项目1235项。在全省率先完成食品药品监管体制改革。深化财税体制改革，推进营业税改增值税试点工作。加强对机关和干部作风、纪律、效能的监督检查，对274个单位的353名责任人进行严肃问责，干部作风明显转变。

2014年主要工作

　　2014年是全面贯彻落实党的十八届三中全会精神、全面深化改革的第一年，也是实施“十二五”规划承前启后的关键一年。根据市委的总体部署，政府工作要全面贯彻落实党的十八大和十八届三中全会精神，改革创新、稳中求进。坚持以经济建设为中心，以改革促发展、促转方式调结构。坚持经济发展与环境、资源、安全相协调，增加经济总量与提高质量并重，强化发展基础，壮大经济实力。坚持实施工业强市、城乡融合和生态立市战略，着力改善民生。强化反腐倡廉建设，深入开展党的群众路线教育实践活动。改进作风，抢抓机遇，狠抓落实，确保深化改革顺利推进、经济持续健康发展、社会大局和谐稳定的良好发展态势。

　　经济社会发展的主要预期目标是：生产总值增长12.5%。全社会固定资产投资增长25%，招商引资到位资金增长26%。工业增加值增长16%。社会消费品零售总额增长15%。公共财政预算收入增长18%。城镇居民人均可支配收入增长13%，农民人均纯收入增长16%。城镇登记失业率控制在4%以内。人口自然增长率控制在7‰以内。城镇化率提高2个百分点。居民消费价格涨幅控制在4%以内。单位生产总值能耗和主要污染物排放量完成省政府下达的控制指标。

　　围绕上述目标，重点做好九个方面的工作：

　　（一）坚持项目投资拉动，着力扩大经济总量。深入实施“项目带动”行动，以“3341”项目工程为总抓手，坚持招商引资与向上争取并重、扩大总量与提高质量并重、加快发展与夯实基础并重，全力推动转型跨越。力争实施500万元以上项目900项以上，亿元以上项目260项以上，确保在建项目规模保持在1000亿元以上。抓好项目落地和组织实施，开工建设干塘至武威增建铁路二线，加快建设民勤县城至红沙岗一级公路、天祝至青海互助二级公路、武威综合客运枢纽中心、武威热电联产、红崖山水库加高扩建、毛藏寺水库等项目，力争石门河调蓄引水、古浪县大靖城区供水、天祝县南阳山片供水工程建成投运；加快建设民勤县百万千瓦级光电基地，建成凉州区百万千瓦级光电、民勤红沙岗百万千瓦级风电基地，力争风光电装机规模累计达到300万千瓦以上；加快内蒙古阿拉善左旗至武威至西宁高速公路民勤至武威段、天祝至景泰二级公路、兰新铁路三四线中川至张掖段项目前期工作，争取立项启动实施。

　　（二）加快培育特色工业体系，着力推动经济转型跨越发展。坚持引项目、找矿藏、建园区、上规模、创品牌、增效益，深入实施工业强市战略，扎实推进“多极突破”行动。围绕培育5个销售收入过百亿元的企业、建设5个销售收入过百亿元的产业园区、打造10个百亿级产业链，培育壮大农产品精深加工、汽车装备进出口、建材工业、特色化工、新能源及装备制造业，加快构建新型工业体系。力争新开工千万元以上工业项目130项，建成120项，完成工业固定资产投资336亿元。用好“中国葡萄酒城”金字招牌，突出抓好以葡萄酒为主的“液体经济”首位产业，力争开工建设青啤40万千升啤酒及配套玻璃瓶、易拉罐项目，加快建设莫高国际葡萄酒城等项目。

　　（三）加快建设城乡融合发展核心区，着力推进以人为核心的城镇化进程。坚持以城乡融合发展理念统领全市总体规划，以城乡融合发展核心区建设带动全面发展。修订完善武威城乡融合发展总体规划、城市总体规划、三县总体发展规划和县城总体规划，编制完成凉州中心城南片区、天祝县城南区控制性详规、凉州区G30沿线新农村建设规划和村镇风貌规划。加快建设干支路网，配套完善沿线公交站点、油气站点、物流节点和公共服务设施，建成核心区三级路网200公里。以金色大道、G30、民武路、民昌路沿线和金强河沿岸为重点，全力推进新型农村社区和中心村建设，改善农村人居环境，力争开工建设300户以上示范点10个。加快推进海藏湖生态治理、天马湖景观治理二期工程、天马湖滨河公园等项目和城市天然气综合利用、德国促进贷款建设武威市城西集中供热工程，启动建设武威市污水处理厂污水再生利用工程，持续推进供热、供水、污水处理及配套管网改造等基础设施建设。开工建设世行贷款城乡融合发展核心区基础设施项目，加大县城和重点小城镇建设力度，加快产业培育和公共服务设施建设，不断增强人口和产业集聚能力。

　　（四）加大生态保护建设力度，着力增强可持续发展支撑能力。深入实施“生态屏障”行动，统筹抓好节水、治沙、造林、防污四个重点，扎实推进石羊河流域后期治理。强化水资源调度管理，大力开展人工增雨雪作业，有效增加自然降水和河流来水量，全面完成石羊河流域后期治理年度任务，地下水开采量控制在4.84亿立方米以内，其中民勤盆地地下水开采量控制在0.86亿立方米以内，确保平水年蔡旗断面过水量达到2.5亿立方米以上。抢抓国家批准建设《甘肃省加快转型发展建设国家生态安全屏障综合试验区总体方案》机遇，争取实施祁连山水源涵养区生态环境保护与综合治理规划。拓宽荒漠野生动物保护新视野，争取实施濒危野生动物民勤青土湖试验性放养基地建设项目。严格落实“五禁”规定，推进石羊河流域防沙治沙及生态恢复、“三北”防护林、退耕还林、生态公益林效益补偿等重点生态项目，建设红水河百里生态经济长廊，加快建设北部防沙治沙生态屏障。完成人工造林50万亩、封育36万亩、义务植树1100万株、通道绿化1000公里。突出工业点源、农业面源、生活污染和水、大气污染综合治理，淘汰“黄标车”，减少机动车尾气排放，加强集中式饮用水源地保护，完成17个污染减排、54个规模化畜禽养殖治理和46个农村环境连片整治项目任务，不断改善环境质量。

　　（五）全面推进主体生产模式，着力增加农民收入。以促进农民增收和确保粮食自给为核心，坚持调结构、促增收、修公路、兴水利、建新房、抓脱贫、美环境，强力推进“设施农牧业+特色林果业”主体生产模式和“贮藏加工+运输销售”营销模式，构建“企业+专业合作组织+基地+农户”的经营模式，走好“规模化、区域性、多品种、高效益”的发展路子。新建设施农牧业15万亩，实现户均2亩棚的目标；新发展特色林果业32万亩。推广种植饲用型甜高粱和紫花苜蓿。落实獭兔产业发展规划，力争当年饲养量达到6000万只。积极培育壮大畜牧养殖企业，建成一批万只羊单位的大型养殖企业。统筹推进“1236”扶贫攻坚和“双联富民”行动，利用国开行贷款和“双联”贷款、“双业”贷款、獭兔养殖贴息贷款，帮助贫困群众发展设施农牧业和特色林果业，力争贫困片区农牧民人均纯收入增幅高于全省平均水平2个百分点以上，减少贫困人口10万人。构建大扶贫格局，加大政策、项目和资金对接争取力度，加快路、水、电、房等基础设施建设，全力实施“下山入川”工程，不断改善贫困群众生产生活条件。抓好全省“美丽乡村”民勤县试点建设。实施能力素质提升工程，力争“两后生”职业技能学历培训达到7000人，劳动力技能培训达到1.1万人以上。加大劳务输转力度，强化信息服务和就业指导，完成劳务输转42万人，实现劳务收入55亿元。

　　（六）加快现代服务业发展，着力打造丝绸之路经济带武威黄金节点。抢抓国家加快向西开放、建设丝绸之路经济带重大机遇，加快建设同威武威煤炭集疏运中心，建成武威保税物流中心、黄羊公铁联运物流中心、万嘉国际广场、武威昊天农产品交易市场暨仓储物流中心等区域性物流基地和跨区域大型商品交易市场，新建和改造提升一批专业批发市场、农村集贸市场、商品配送中心，努力将武威建成丝绸之路经济带黄金节点。推进华夏文明传承创新区武威项目区建设，大力发展文化旅游业，抓好博物馆、影剧院、图书馆等重点项目，发展公益性文化事业。积极推进天梯山历史文化旅游景区、天祝石门沟景区等重点项目建设，加快培育建设武（威）金（昌）综合旅游景区，创建A级景区和星级饭店。办好“中国葡萄酒城第二届葡萄酒节”、第四届“敦煌行·丝绸之路国际旅游节”武威特色节会活动。

　　（七）全面协调发展社会各项事业，着力改善民生。把改善民生作为一切工作的出发点和落脚点，不断提高人民群众生活质量。扎实推进“全民创业行动”，促进农民就近就地就业，实施高校毕业生就业扶持工程，鼓励支持大学生回乡自主创业，城镇新增就业1.8万人，应届普通高校毕业生就业率达到85%以上。加快各类保障性住房和新型农村社区建设，改善城乡居民住房条件。基础教育重点实施好义务教育标准化学校建设、学前教育推进、特困地区普通高中建设，优化配置教育资源，大力推行素质教育，全面提高教育质量；职业教育重点加强基础能力工程建设，争取申办凉州职业学院，加快武威职业学院一期扩建项目建设，确保武威职业学院“国家示范性高等职业学院建设计划”骨干高职院校建设项目顺利通过验收。着力办好高等教育，不断扩大招生规模。加快省级创新型试点城市、民生科技试点市和甘肃武威国家农业科技园区建设，促进科技成果转化，提升区域创新能力。加快中科院近物所武威核技术利用综合产业园建设，全面带动与重离子相关的产业发展。建成武威重离子治疗肿瘤中心暨荣华颐养园，完成市人民医院整体搬迁项目一期工程，开工建设市中医院综合服务大楼，启动实施天祝县妇幼保健院搬迁项目。继续抓好武威市恶性肿瘤高发区防控模式示范项目、乙肝防治示范区建设。扶持中医药和民族医药事业发展。稳定低生育水平，提高人口素质，严格控制人口自然增长率。大力开展全民健身活动，不断提高人民群众的健康水平。

　　按照量力而行、尽力而为的原则，集中力量办好10件实事：1.解决20万农村人口饮水安全问题；2.新建改建农村公路650公里；3.加快推进高深山区农牧民“下山入川”工程，完成移民2万人；4.全市农村危房改造、新房建设5万户；5.在市区中小学人流密集路段规划建设人行过街天桥3座；6.新改建中小学校舍16万平方米、幼儿园20所，在城乡融合发展核心区新改建学校15所；7.改建2所妇幼保健院、2个急救中心、29个乡镇卫生院、57个村卫生室；8.城乡低保标准提高15%，农村五保供养标准提高300元；9.为全市一级重度残疾人发放护理补贴；10.建设社会治安视频监控及智能交通管理项目二期工程。

　　（八）创新社会治理，着力维护和谐稳定大局。深入实施“和谐构建行动”，全面推进平安武威建设。坚持用群众工作统揽信访工作，加大矛盾纠纷排查调处和信访积案化解力度，靠实信访工作责任，把各种不稳定因素化解在基层和萌芽状态。坚持依法治理，完善人民调解、行政调解、司法调解联动工作体系，探索社会服务管理工作新模式，加强新型农村社区管理，“下山入川”群众以移入地管理为主，确保搬得出、稳得住、能发展、可致富。加强应急管理体系建设，提高综合防范和抵御灾害能力。抓好国家粮食收储供应安全保障工程。强化食品药品及产品质量安全监管，建立最严格的覆盖生产、流通、消费全过程的监管制度，保障人民群众身体健康。严格落实安全生产责任制，坚守“发展不能以牺牲人的生命为代价”这条红线，深入开展安全生产大检查，坚决整治各类事故隐患，严厉打击非法违法生产经营建设行为，杜绝重特大安全生产事故发生。坚持改革、发展、稳定三位一体，认真落实支持藏区发展的政策措施，广泛开展民族团结进步创建活动，依法加强民族宗教事务管理，扎实推进“两个共同”示范县建设，打牢藏区稳定基础，确保天祝藏区和谐稳定发展。深入推进“六五”普法，加快依法治市进程。完善政府调控和市场调节机制，加强价格监管，保持物价总水平基本稳定。

　　（九）深化改革开放，着力增强转型跨越发展的动力和活力。认真贯彻落实党的十八届三中全会和省委十二届六次全委（扩大）会议、市委三届六次全委（扩大）会议的决策部署，胆子要大、步子要稳，在坚持基本经济制度、完善发展体制机制上取得突破；在简政放权、转变政府职能上取得突破；在夯实发展基础、培育壮大支柱产业上取得突破；在坚持做大经济总量、提高经济质量上取得突破；在协调发展社会事业、全面改善民生上取得突破；在实施城乡融合发展、推进新型城镇化进程上取得突破；在创新投融资机制、强化金融保障服务上取得突破。坚持“两先一放一管”，把中央和省委、市委已经明确的改革事项先做起来，把已经看准的事情先干起来，加快改革发展步伐。一是管好用活国土资源。坚持“以土地换项目”、“以资源换项目”。统筹利用城乡土地，建立投资、用地协调机制，将凉州城区内土地审批管理等事权下放凉州区。盘活存量土地，提高土地集约利用效益。用足用活用好石羊河流域重点治理压减耕地特殊政策，全力保障重点建设项目和工业用地。合理开发利用矿产资源，打破行政区划界限，建设跨区域开发区。完成农村宅基地、集体建设用地使用权登记颁证工作，为农村集体建设用地流转、土地抵押担保融资创造条件，鼓励支持农民以承包经营权入股发展农业产业化经营，增加农民财产性收入。二是积极探索推进林业综合改革。深化集体林权制度和国有林场改革，建立健全严格的森林资源保护制度，不断完善治沙生态林承包经营机制，探索建立以特色林果为主体、复合经营、生态经济的沙产业发展模式。三是理顺市区城建管理体制。将凉州城区城市建设和管理等事权下放凉州区。在城市管理领域开展相对集中行政执法权工作，提高城市管理水平。探索实施“园区带乡镇”模式，推行工业园区与乡镇“两块牌子、一套班子、以园区为主”的管理体制，带动和催生一批各具特色、功能完善的中小城镇，加快城镇化进程。四是推进财税管理改革。深化部门预算管理改革，实施全面规范、公开透明的预算制度。加强税收征管，落实税收政策，积极培植税源。调整优化支出结构，把有限的资金用于稳增长、调结构、惠民生，集中财力办大事。五是深化教育领域综合改革。突出促进教育公平，健全家庭经济困难学生资助体系，构建利用信息化手段扩大优质教育资源覆盖面的有效机制，统筹城乡义务教育资源均衡配置，逐步缩小城乡、校际差距。六是深化科技体制改革。围绕重点领域和产业发展，建立产学研政协同创新机制，强化企业在技术创新中的主体地位，激发创新活力，加强知识产权应用和保护，提高科技支撑能力。七是深化医药卫生体制改革。巩固完善基本药物制度，推进公立医院改革，加强医疗卫生信息化建设，完善药品价格形成机制。

　　加强区域合作，扩大开放开发，鼓励支持各区县按照“优势互补、互利共赢”的原则，着力构建多层次、宽领域、广依托、全方位的开放开发格局。坚持“重商、亲商、安商、富商”理念，着力营造优质高效的政务环境、公平公正的社会环境和“能赚钱、不受气、条件好”的招商引资环境，吸引更多战略投资者，共同建设幸福美好新武威！
